# Differences in radiation dose for computed tomography of the brain among pediatric patients at the emergency departments: an observational study

**DOI:** 10.1186/s12873-021-00502-7

**Published:** 2021-09-22

**Authors:** Xi Min Tan, Mohammad Taufik Bin Mohamed Shah, Shu-Ling Chong, Yong-Kwang Gene Ong, Peck Har Ang, Nur Diana Bte Zakaria, Khai Pin Lee, Jen Heng Pek

**Affiliations:** 1 Yong Loo Lin School of Medicine, National University of Singapore, 10 Medical Dr, Singapore, 117597 Singapore; 2 Department of Radiology, Sengkang General Hospital, 110 Sengkang E Way, Singapore, 544886 Singapore; 3 Department of Emergency Medicine, KK Women’s and Children’s Hospital, 100 Bukit Timah Rd, Singapore, 229899 Singapore; 4 Accident and Emergency Department, Changi General Hospital, 2 Simei Street 3, Singapore, 529889 Singapore; 5 Department of Emergency Medicine, Singapore General Hospital, Outram Rd, Singapore, 169608 Singapore; 6 Department of Emergency Medicine, Sengkang General Hospital, 110 Sengkang E Way, Singapore, 544886 Singapore

**Keywords:** Computed tomography, Emergency, Pediatric, Radiation

## Abstract

**Background:**

Computed tomography (CT) is associated with a risk of cancer development. Strategies to reduce radiation doses vary between centers. We compared radiation doses of CT brain studies between pediatric and general emergency departments (EDs), and determine the proportion studies performed within the reference levels recommended by the International Commission on Radiological Protection (ICRP).

**Methods:**

A retrospective review was carried out in a healthcare network consisting of one pediatric ED and three general hospital EDs. Pediatric patients less than 16 years old with CT brain studies performed between 1 January 2015 and 31 December 2018 were included. Information on demographic, diagnosis, volume-averaged computed-tomography dose index and dose length product (DLP) were collected. Effective dose was then calculated from DLP using conversion factors, termed k-coefficients which were derived using a 16 cm head CT dose phantom.

**Results:**

Four hundred and seventy-nine CT brain studies were performed – 379 (79.1%) at the pediatric ED. Seizure (149, 31.1%), head injury (147, 30.7%) and altered mental status (44, 9.2%) were the top three ED diagnoses. The median effective dose estimates were higher in general than pediatric EDs, particularly for those aged > 3 to ≤6 years old [1.57 mSv (IQR 1.42–1.79) versus 1.93 mSv (IQR 1.51–2.28), *p* = 0.047], > 6 to ≤10 years old [1.43 mSv (IQR 1.27–1.67) versus 1.94 mSv (IQR 1.61–2.59), *p* = 0.002) and > 10 years old (1.68 mSv (IQR 1.32–1.72) versus 2.03 mSv (IQR 1.58–2.88), *p* < 0.001). Overall, 233 (48.6%) and 13 (2.7%) studies were within the reference levels recommended by ICRP 60 and 103 respectively.

**Conclusions:**

Radiation doses for CT brain studies were significantly higher at general EDs and less than half of the studies were within the reference levels recommended by ICRP. The development of diagnostic reference levels (DRLs) as a benchmark and clinical justification for performing CT studies can help reduce the radiation risks in the pediatric population.

**Supplementary Information:**

The online version contains supplementary material available at 10.1186/s12873-021-00502-7.

## Background

Computed tomography (CT) is a valuable diagnostic tool in the Emergency Department (ED) [1]. However, it involves the use of ionizing radiation at much higher doses as compared to other diagnostic imaging tests such as x-rays [2]. Given the increased utilisation of CT in recent years, concerns have therefore been raised about the potential for developing malignancies resulting from exposure to ionizing radiation during CT studies [1, 3–5]. The pediatric population is particularly susceptible due to their radiosensitive organ tissues and long lifespan after radiation exposure, with an increased incidence of radiation-induced cancers such as leukaemia and brain tumours [1, 6–15].

Previously, radiation doses were similar for pediatric and adult CT techniques – despite the smaller body habitus and increased radiosensitivity of children [16]. Recommendations for reducing ionizing radiation dose for pediatric patients started to emerge in the early 2000s, led by the “As Low As Reasonably Achievable” and “Image Gently” campaigns to raise awareness about the risks associated with CT studies and propose protocols to reduce the radiation dose for children [13, 16–19]. However, literature on pediatric multi-slice CT studies reported an effective dose of about 4 mSv for CT brain examinations, as opposed to an effective dose of 1 to 2 mSv in adults, suggesting that a gap still exists in clinical practice [20, 21]. In addition, effective doses in pediatric CT head studies can vary across healthcare facilities, particularly between institutions with dedicated pediatric services and those without [22–28]. Reasons for such disparities include inappropriate clinical justification; patient-related attributes like size, weight, motion, and complexity of medical condition; as well as technical factors like a CT scanner’s acquisition parameters, and the level of training or experience of the CT technologist and radiologist [23, 29]. As such, there is a continual need to push for appropriate justification of pediatric CT studies with optimisation of radiation dose used [30, 31].

To this end, a landmark development was when the American Association of Physicists in Medicine (AAPM) established the size-specific dose estimate (SSDE) which takes into consideration the patient’s size, and allows for optimisation of radiation dose without sacrificing image quality [32–34]. Also, the International Commission on Radiological Protection (ICRP) has since updated its age and region-specific effective dose limits from the previous 1990 Publication 60 recommendations to the current 2007 Publication 103, based on the latest available scientific information of the biology and physics of radiation exposure [35, 36]. Finally, the establishment of a CT dose diagnostic reference level (DRL), defined as a radiation dose level to identify situations where the patient dose or administered activity is unusually high, further helps with dose optimisation [37]. In healthcare practices, DRLs help prevent radiation exposures that do not provide additional information for patient clinical management [38]. They also help guide radiology departments review their practices when patient doses significantly deviate from the DRL, and allow comparisons between institutions for quality control purposes. At the time of writing, there is no established national or local DRL guidance for pediatric CT head examinations in Singapore.

Therefore, our primary objective was to compare radiation doses used for pediatric CT brain studies between pediatric and general EDs of a healthcare network in Singapore, and determine the proportion of CT brain studies performed within the reference level recommended by ICRP [35, 36, 39]. (Supplementary Materials). Our secondary objective was to propose a DRL for pediatric CT brain studies performed in the EDs. We hypothesize that for pediatric CT brain studies, the median effective radiation dose administered in general EDs is higher compared to the pediatric ED, and a greater proportion of CT studies is within ICRP 60 and 103 recommendations at pediatric than general EDs. The findings from this work can drive quality improvement initiatives and ultimately, it is about making CT imaging safe for every pediatric patient in the ED.

## Methods

### Study setting

The public healthcare institutions in Singapore are divided into three healthcare networks, with each serving a specific geographical location. Our healthcare network is the largest and consists of (1) a pediatric tertiary hospital with a pediatric ED and both inpatient and outpatient pediatric services; (2) three adult tertiary hospitals with general EDs and no inpatient or outpatient pediatric service. The pediatric ED is staffed by pediatric emergency physicians while the general EDs are staffed by emergency physicians who have variable experience in pediatric emergency care.

### Study design

A retrospective study was carried out involving pediatric patients, defined by age less than 16 years old, who attended the EDs from 1 January 2015 to 31 December 2018 and had a CT brain study performed. Data was collected using a standardized form by assessing the electronic medical records of the ED visit. Information on demographic, diagnosis and radiation dose in terms of volume-averaged computed-tomography dose index (CTDI_vol_) and dose length product (DLP) were obtained and analysed. All CT brain studies will be analysed, including those repeated in the same ED visit.

This study was approved by Institutional Review Board at SingHealth, with waiver of informed consent.

### CT scanner parameters

Across all EDs, CT brain studies were performed using one of the following three CT scanners: Toshiba Aquilion (Toshiba Medical Systems, Tokyo, Japan), Toshiba Aquilion Prime (Toshiba Medical Systems, Tokyo, Japan) or Siemens Somatom Force (Siemens Healthcare, Germany).

### Radiation dose parameters

Dose indicators of CTDI_vol_ and DLP were obtained. CTDI_vol_ reflects the mean absorbed radiation within the scan volume based upon standardized CTDI phantoms. For CT brain studies, CTDI quantification is based on a 16 cm diameter Plexiglas phantom [21]. DLP, determined by multiplying the CTDI_vol_ (in mGy) by scan length (in cm), is a measure of a CT scanner’s radiation output/exposure along a patient’s long axis (in mGy-cm) and provides an estimate of the total energy delivered to the CTDI Plexiglas phantom – consequently, the scan length may be estimated by dividing the DLP by the CTDI_vol_ [21].

Effective dose, in mSv, was calculated from DLP by multiplying DLP with age- and region-specific conversion factors, termed k-coefficients (in mSv/(mGy-cm)), recommended by the ICRP 60 and 103 [35, 36, 39]. (Supplementary Materials).

### Data analysis

SPSS version 22 (SPSS, Chicago, IL) was used to perform statistical analysis. Frequencies with percentages were used to present categorical data. Mean ± standard deviation (SD) or median (interquartile range, IQR) was used for continuous data depending on normality. Patients were classified into the following five age-group categories – ≤6 months, > 6 months to ≤3 years, > 3 years to ≤6 years, > 6 years to ≤10 years, > 10 years – to compare their effective doses and with the reference levels recommended by ICRP. Chi-square test or Fisher’s exact test was used for association between categorical data. Student’s t-test or Mann-Whitney U test was used for association between continuous data depending on normality. Statistical significance was taken at p less than 0.050.

## Results

### Demographics

A total of 479 pediatric patients had CT brain studies performed over the study period – 379 (79.1%) in the pediatric ED and 100 (20.9%) in the three general EDs. All of the CT brain studies were non-contrasted. There was no patient with multiple CT brain studies in a single ED visit. The median age of the patients across the four EDs was 7 years (IQR 3 to 12) and there were 290 (60.5%) males. Seizure (149, 31.1%), head injury (147, 30.7%) and altered mental status (44, 9.2%) were the top three ED diagnoses for patients requiring CT brain studies in the EDs (Table 1).

**Table 1:** Patient Demographics

	Overall (*n* = 479)	Pediatric ED (*n* = 379)	General EDs (*n* = 100)	*p*-value
Median Age in Years (Interquartile Range)	7 (3 to 12)	6 (2 to 10)	13 (10 to 15)	**< 0.001**
Gender, n (%)		`		0.426
Male	290 (60.5)	226 (59.6)	64 (64.0)	
Diagnosis, n (%)				**< 0.001**
Seizure	149 (31.1)	127 (33.5)	22 (22.0)	
Head Injury	147 (30.7)	105 (27.7)	42 (42.0)	
Altered Mental Status	44 (9.2)	36 (9.5)	8 (8.0)	
Stroke	34 (7.1)	34 (9.0)	0 (0)	
Headache, not specified	22 (4.6)	9 (2.4)	13 (13.0)	
Meningoencephalitis	21 (4.4)	18 (4.7)	3 (3.0)	
Intracranial Neoplasm	11 (2.3)	11 (2.9)	0 (0)	
Cardiac Arrest	9 (1.9)	4 (1.1)	5 (5.0)	
Syncope	9 (1.9)	5 (1.3)	4 (4.0)	
Intracranial Haemorrhage	6 (1.3)	4 (1.1)	2 (2.0)	
Ventriculoperitoneal Shunt-related	3 (0.6)	3 (0.8)	0 (0)	
Others	24 (5.0)	23 (6.1)	1 (1.0)	

### CTDI_vol_, DLP and effective dose for CT brain studies

Table 2 shows the parameters of the CT scanners used at the pediatric and general EDs.

**Table 2:** Parameters of CT Scanners At Pediatric and General EDs

Parameters	Pediatric ED	General EDs
Tube Potential, kV	100, 120	100,120
Median Tube Current - Exposure Time Product (IQR), mAs		
≤ 6 months	148.50 (135.25–184.00)	263.00 (−)
> 6 months to ≤3 years	176.00 (156.00–210.00)	288.00 (257.25–300.00)
> 3 years to ≤6 years	196.00 (181.75–239.00)	294.00 (229.50–336.00)
> 6 years to ≤10 years	244.00 (192.50–260.00)	300.00 (250.00–312.00)
> 10 years	204.00 (187.00–264.00)	320.00 (238.00–360.00)
Median Scan Range (IQR), cm		
≤ 6 months	16.32 (15.14–17.36)	12.93 (−)
> 6 months to ≤3 years	18.20 (16.91–19.50)	17.40 (10.89–27.74)
> 3 years to ≤6 years	18.63 (18.00–19.74)	13.41 (11.58–17.24)
> 6 years to ≤10 years	19.17 (18.49–20.32)	17.16 (15.02–18.60)
> 10 years	19.60 (18.78–20.63)	18.49 (16.78–19.39)
Pitch Value	0.55–1.55	0.5–1.55
Rotation Time, seconds	0.28–1.0	0.5–1.0
Collimation, mm		
Siemens	128 × 0.6, 192 × 0.6	128 × 0.6, 192 × 0.6
Toshiba	40 × 0.6	40 × 0.6
Reconstruction Technique	Iterative	Iterative
Section Thickness, mm	3	3

There were significant differences in both median CTDI_vol_ and DLP between the pediatric ED and general EDs in the > 3 to ≤6 years, > 6 to ≤10 years and > 10 years age groups. (Supplementary Materials) The median effective dose for all CT brain studies was 1.68 mSv (IQR 1.43 to 2.10 mSv) which was slightly more than half a year’s worth of background radiation exposure in the United States (3.1 mSv/year) [40]. Across all age groups, there was a trend towards greater median effective doses for CT brain studies performed in the general EDs as compared to the pediatric ED. In particular, significant differences in the CT brain median effective doses were found in the > 3 to ≤6 years (*p* = 0.047), > 6 to ≤10 years (*p* = 0.002) and > 10 years (*p* < 0.001) age groups (Table 3).

**Table 3:** Comparison of Effective Dose for CT Brain Studies Between Pediatric and General EDs

Age Group	Overall (*n* = 479)		Pediatric ED (*n* = 379)		General EDs (*n* = 100)		*p*-value
	Median Effective Dose (IQR), mSv	n (%)	Median Effective Dose (IQR), mSv	n (%)	Median Effective Dose (IQR), mSv	n (%)	
≤6 months	1.84 (1.60–2.65)	22 (4.6)	1.79 (1.59–2.39)	20 (5.3)	2.95 (−)	2 (2.0)	0.052
> 6 months to ≤3 years	1.97 (1.67–2.29)	118 (24.6)	1.97 (1.67–2.26)	112 (29.6)	2.57 (1.76–4.01)	6 (6.0)	0.063
> 3 years to ≤6 years	1.57 (1.42–1.57)	87 (18.2)	1.57 (1.42–1.79)	82 (21.6)	1.93 (1.51–2.28)	5 (5.0)	**0.047**
> 6 years to ≤10 years	1.48 (1.30–1.76)	88 (18.4)	1.43 (1.27–1.67)	72 (19.0)	1.94 (1.61–2.59)	16 (16.0)	**0.002**
> 10 years	1.63 (1.38–2.10)	164 (34.2)	1.68 (1.32–1.72)	93 (24.5)	2.03 (1.58–2.88)	71 (71.0)	**< 0.001**

### Proportion of CT brain studies with effective doses within the reference levels recommended by ICRP 60 and 103

Two hundred and thirty-three (48.6%) of all the CT brain studies done across both pediatric and general EDs fell within the reference levels recommended by ICRP 60. The proportion is significantly higher in the pediatric ED where 216 (45.1%) were within ICRP 60 definition, compared to 13 (2.7%) in general EDs (*p* < 0.001). However, when the newer ICRP 103 definition was used, only 13 (2.7%) of all CT brain imaging studies were within the reference levels, all of which were in the pediatric ED (Table 4).

**Table 4:** CT Brain Studies with Effective Doses within the Reference Levels Recommended by ICRP 60 and 103

Age Group	ICRP 60		ICRP 103	
	Pediatric ED (*n* = 379)	General EDs (*n* = 100)	Pediatric ED (*n* = 379)	General EDs (*n* = 100)
≤6 months	15 (4.0)	0 (0)	6 (1.6)	0 (0)
> 6 months to ≤3 years	47 (12.4)	2 (2.0)	3 (0.8)	0 (0)
> 3 years to ≤6 years	53 (14.0)	2 (2.0)	1 (0.3)	0 (0)
> 6 years to ≤10 years	62 (16.4)	8 (8.0)	0 (0)	0 (0)
> 10 years	39 (10.3)	5 (5.0)	3 (0.8)	0 (0)

### Proposal of a local diagnostic reference level

Based on the recommendations of National Council on Radiation Protection and Measurements (NCRP) Report 172 and ICRP 103, the median CTDI_vol_ and DLP values for each age group may be defined as the local DRL. These proposed DRLs from our study are displayed in Table 5.

**Table 5:** Proposed Local Diagnostic Reference Level for Pediatric CT Brain Studies

Age Group	Pediatric ED		General EDs	
	CTDI_vol_	DLP	CTDI_vol_	DLP
≤6 months	19	309	27	335
> 6 months to ≤3 years	22	405	29	598
> 3 years to ≤6 years	25	448	40	552
> 6 years to ≤10 years	26	494	43	654
> 10 years	29	571	50	827

## Discussion

CT brain studies remain a sensitive and readily available tool for many diagnostic dilemmas in the ED. CT has the ability to produce images in a quick, non-invasive and reliable manner, making it the best imaging option at the ED over alternative imaging modalities such as paediatric cranial ultrasound or magnetic resonance imaging of the brain. Ultrasound is constrained by the fact that it can only be performed prior to fontanelle fusion in neonates or infants and magnetic resonance imaging requires patients to be absolutely still for a prolonged duration of which the pediatric population may not be able to comply with. CT, however, delivers considerably higher radiation doses than these alternatives. Therefore, minimizing radiation doses during CT brain scans remains an important quality measure in all hospitals.

We found that the effective doses of radiation for CT brain studies were higher in the general EDs than the pediatric ED. By comparing effective doses to the published ICRP 60 and 103 definitions, we found that even in pediatric institutions, less than 50% met the ICRP 60 definitions and less than 5% met the newer ICRP 103 definitions. Therefore, moving forward, we have proposed a local DRL based on the median CTDI_vol_ and DLP values for each age group to improve the safety and quality of CT brain studies performed in pediatric patients at our healthcare network.

The term ‘effective dose’, used by health practitioners worldwide, is regarded as the most appropriate dose descriptor to quantify and communicate the stochastic risks associated with diagnostic procedures involving ionizing radiation [41]. It takes into account the relative sensitivity of a person’s irradiated organs, translating it into a quantifiable estimate of an individual’s biologic detriment (e.g., carcinogenesis) – this is referred to as ‘normalized’ effective dose [35]. In neonates and children, an inherently higher sensitivity to the effects of ionizing radiation can result in doubling of the effective dose delivered to the irradiated anatomic site (e.g., brain) compared to an adult [10, 42].

The age-stratified effective doses of CT brain studies were higher in the younger age groups compared to older age groups; a finding in line with recent publications which supports the inverse relationship between effective dose and age [43, 44]. However, the effective doses for pediatric CT brain studies performed by general EDs were higher, reaching statistical significance in those aged three and above, adding further evidence to the literature that non-dedicated pediatric centers exposed patients to higher radiation doses during CT studies [45–47]. In addition, it is concerning that there is a wider variation across effective doses in the general EDs and a higher proportion of studies exceeding the dose recommendations of ICRP 60. As ICRP 103 conversion coefficients are deemed to be more accurate for children of different ages, it is therefore alarming that when benchmarked against the ICRP 103 effective dose recommendations, none of the CT brain studies at the general EDs was within the reference levels [48].

For CT studies, effective dose is directly proportional to the quantity of energy a scanner emits, quantified by CTDI_vol_ and DLP. While DLP values help determine estimations of effective dose using ICRP 103 age-based conversion coefficients, CTDI_vol_, however, is the CT index that ‘best represents the average dose at a particular point within a scan volume’. [49] The CTDI_vol_ is dependent on several key acquisition parameters such as tube current and scanning rotation time (mAs), tube potential, pitch setting and detector configuration, as well as reconstruction technique and slice thickness; all ultimately contribute to patient’s effective dose [50, 51]. Differences in acquisition parameters of the CT scanners at the EDs may account for the lower doses of radiation at the pediatric ED compared to general EDs. Specifically, general EDs had a higher mAs in all age-groups, pediatric ED had a lower gantry rotation time of 0.28 s, and general EDs had a greater proportion of younger children whose images are acquired at a tube potential of 120 kV voltage setting.

However, acquisition parameters of the CT scanners are not the only reasons for the higher doses used for CT brain studies at the general EDs. While the use of optimal scanning protocols involving pediatric patients may lead to lower radiation doses during CT brain studies, the pediatric protocols are not the default setting at the general EDs, thereby necessitating a switch by the radiographer when the study is being performed [52]. Furthermore, radiologists at the general EDs may be less experienced and familiar with CT studies in pediatric patients, therefore making modifications to protocols in place by increasing doses of radiation to decrease image noise for better diagnostic accuracy [26, 28]. All these deviations can contribute to the higher radiation doses for CT brain studies at the general EDs.

Optimal radiation doses should be established by regulatory authorities as national DRLs using national survey, or healthcare facilities as local DRLs using current practice [39]. However, both national and local DRLs are lacking in Singapore. In our study, we proposed local DRLs based on the median CTDI_vol_ and DLP at pediatric and general EDs. In doing so, we hope to call to attention the need for better regulation of radiation exposure from CT studies in pediatric patients. When compared to European guidelines, the local DRLs were higher, especially those for general EDs. (Supplementary Materials).

This study is an initial but critical step towards understanding where we currently stand in terms of pediatric CT dose levels and how it varies across the EDs in our healthcare network. The processes involved in determining radiation dose estimates, including local DRLs, have provided us with an evaluation framework and tools for optimizing doses for pediatric CT brain studies performed in our EDs. At the same time, they have shown us the potential challenges of establishing consensual DRL for pediatric CT studies. The local DRL values in this review were well below those reported by our American and European counterparts, which is encouraging. However, dose variations found between pediatric and non-pediatric EDs within our healthcare network are troubling. Nonetheless, we hope our findings serve as a stimulus for a concerted nationwide and unified approach to pediatric CT dose optimisation, involving physicians, allied health professionals, and patients.

For institutions, we propose having an active approach to educate and raise awareness among healthcare workers beyond ED and radiology staff as part of our efforts to reduce radiation doses in pediatric patients. Implementation of quality control measures to ensure competence when dealing with pediatric patients and monitoring compliance to departmental pediatric protocols for scanning should be considered, especially in centers without dedicated pediatric services. Finally, it is our responsibility to ensure that pediatric CT studies are clinically indicated, and that their acquisition techniques and protocols are optimized. Frequent reviews of pediatric CT protocols to prevent unnecessary radiation exposure to our younger population from routine day-to-day medical practice are highly encouraged.

### Limitations

This study has several limitations. Firstly, this study was conducted in a single healthcare network consisting of a pediatric ED and three general EDs in Singapore. All four centers are tertiary hospitals and academic centers. As EDs in other settings may have different practices, a collaboration involving various institutions from multiple countries would be able to provide a better representation of how radiation doses used for CT studies differed between pediatric and general EDs. Next, we only evaluated non contrasted CT brain studies instead of including all CT studies as the number of CT studies involving contrast and/or other body regions were performed infrequently, leading to inadequate numbers for statistical power and valid comparison of radiation doses used between pediatric and general EDs. We also did not evaluate the indications for CT and the number of CT brain studies with positive or clinically important findings.

In determining the effective dose estimates for pediatric CT brain, we used scanner-derived parameters, as well as age- and tube potential-based conversion coefficients recommended by ICRP 103. CTDI_vol_ provides estimates of radiation output doses based on similar attenuating objects and do not take into account those substantially different in terms of size or shape, particularly in children where significant differences in size is often encountered. Other methods including scanner-specific effective dose estimates and size-specific dose estimates are available. Consequently, patients in this review could have been categorized in other ways such as size (e.g., effective head diameter). Furthermore, we did not evaluate the quality of images as this would have improved our understanding of what constitutes image diagnostic adequacy and acceptability across a spectrum of pediatric radiation dose metrics. Hence, future studies should consider including quantitative and qualitative evaluations of diagnostic image quality for completeness. Lastly, we did not study the long term effects of the higher radiation doses used at the general EDs on the subsequent development of radiation-induced cancers.

## Conclusions

CT brain remains an important diagnostic imaging tool that is not easily substituted by alternative imaging modalities. When performed for appropriate indications with optimized technical parameters, the value of the information obtained far exceeds the stochastic risks. However, as general EDs have a tendency to administer higher radiation dose during CT brain study compared to pediatric ED, there must be strategies in place to justify and optimize the use of CT study by adopting imaging protocols with reduced radiation doses based on national or local standards. This will allow for better quality management in pediatric CT imaging, so that radiation exposure can be kept “As Low As Reasonably Achievable” and every pediatric patient can be “Image(d) Gently” in the ED.

## Supplementary Information

**Additional file 1.**

## Data Availability

The datasets generated and analyzed during the current study are not publicly available as per guidelines by Institutional Review Board at SingHealth, Singapore but may be available from the corresponding author on reasonable request.

## Abbreviations

AAPM: American Association of Physicists in Medicine
CTDI_vol_: Volume-averaged computed-tomography dose index
DLP: Dose-length product
DRL: Diagnostic reference levels
ED: Emergency Department
ICRP: International Commission on Radiological Protection
IQR: Interquartile range
SD: Standard deviation
SSDE: Size-specific dose estimate
