# Supplementary material for: Differences in radiation dose for computed tomography of the brain among pediatric patients at the emergency departments: an observational study
Source: BMC Emerg Med. 2021 Sep 22;21:106. doi: 10.1186/s12873-021-00502-7 (PMC8456576; doi:10.1186/s12873-021-00502-7)
Supplement: Supplementary file 1 — Additional file 1. [file 12873_2021_502_MOESM1_ESM.docx]

**Differences In Radiation Dose for Computed Tomography of the Brain Among Pediatric Patients At The Emergency Departments: An Observational Study**

**Author List:**

Xi Min Tan

Mohammad Taufik Bin Mohamed Shah

Shu-Ling Chong

Yong-Kwang Gene Ong

Peck Har Ang

Nur Diana Bte Zakaria

Khai Pin Lee

Jen Heng Pek

**Table. Reference Levels of Effective Dose for a Typical CT Brain Study According to ICRP 60 and 103**

| Age Group | ICRP 60 Effective Dose  (mSv) | ICRP 103 Effective Dose  (mSv) |
| --- | --- | --- |
| ≤6 months | 2.4 | 1.6 |
| >6 months to ≤3 years | 1.9 | 1.3 |
| >3 years to ≤6 years | 1.7 | 1.2 |
| >6 years to ≤10years | 1.9 | 1.3 |
| >10 years | 1.4 | 1.0 |

**Table. K-coefficients for Pediatric CT Brain As Recommended by ICRP 60 and 103**

| Age Group | Tube Voltage (kV) | K-coefficients (mSv/mGycm) | |
| --- | --- | --- | --- |
|  |  | ICRP 60 | ICRP 103 |
| ≤1 year | 100 | 0.0080 | 0.0088 |
|  | 120 | 0.0077 | 0.0085 |
| >1 year to ≤5 years | 100 | 0.0049 | 0.0054 |
|  | 120 | 0.0047 | 0.0053 |
| >5 years to ≤10 years | 100 | 0.0031 | 0.0035 |
|  | 120 | 0.0031 | 0.0035 |
| >10 years to ≤16 years | 100 | 0.0023 | 0.0027 |
|  | 120 | 0.0023 | 0.0027 |
| >16 years | 100 | 0.0015 | 0.0019 |
|  | 120 | 0.0016 | 0.0019 |

**Table. Comparison of CTDI_vol_ and DLP for CT Brain Studies Between Pediatric and General EDs**

| Age Group | Median CTDI_vol_ (IQR), mGy | | | | | Median DLP (IQR), mGy.cm | | | | |
| --- | --- | --- | --- | --- | --- | --- | --- | --- | --- | --- |
|  | Pediatric ED | n (%) | General EDs | n (%) | p-value | Pediatric ED | n (%) | General EDs | n (%) | p-value |
| ≤6 months | 18.98  (17.55-19.93) | 20 (5.3) | 26.75  (-) | 2 (2.0) | 0.052 | 308.50  (276.25-335.90) | 20 (5.3) | 335.00 | 2 (2.0) | 0.361 |
| >6 months to ≤3 years | 22.10  (20.77-23.73) | 112 (29.6) | 28.70  (18.92-49.30) | 6 (6.0) | 0.140 | 404.50  (354.78-455.15) | 112 (29.6) | 597.50  (327.25-748.25) | 6 (6.0) | 0.237 |
| >3 years to ≤6 years | 24.63  (22.31-26.66) | 82 (21.6) | 39.50  (27.53-49.60) | 5 (5.0) | **<0.001** | 448.00  (404.50-512.70) | 82 (21.6) | 551.50  (433.00-652.25) | 5 (5.0) | **0.047** |
| >6 years to ≤10years | 26.15  (24.24-28.53) | 72 (19.0) | 42.79  (31.55-50.58) | 16 (16.0) | **<0.001** | 494.25  (455.25-566.25) | 72 (19.0) | 653.75  (542.75-898.60) | 16 (16.0) | **<0.001** |
| >10 years | 28.80  (26.60-33.21) | 93 (24.5) | 49.30  (43.84-57.60) | 71 (71.0) | **<0.001** | 571.00  (504.50-678.00) | 93 (24.5) | 827.00  (724.30-1100.00) | 71 (71.0) | **<0.001** |

**Table. Comparison of Local Diagnostic Reference Levels with European Guidelines**

| Age Group | European Guidelines | | Pediatric ED | | General EDs | |
| --- | --- | --- | --- | --- | --- | --- |
|  | CTDI_vol_ | DLP | CTDI_vol_ | DLP | CTDI_vol_ | DLP |
| ≤3 months | 24 | 300 | 19 | 311 | 27 | 335 |
| >3 months to ≤12 months | 28 | 385 | 20 | 341 | - | - |
| >1 year to  ≤6 years | 40 | 505 | 23 | 521 | 31 | 528 |
| >6 years | 50 | 650 | 27 | 468 | 48 | 804 |
